# Synergistic effects of selective inhibitors targeting the PI3K/AKT/mTOR pathway or NUP214-ABL1 fusion protein in human Acute Lymphoblastic Leukemia

**DOI:** 10.18632/oncotarget.13035

**Published:** 2016-11-03

**Authors:** Carolina Simioni, Simona Ultimo, Alberto M. Martelli, Giorgio Zauli, Daniela Milani, James A. McCubrey, Silvano Capitani, Luca M. Neri

**Affiliations:** ^1^ Department of Morphology, Surgery and Experimental Medicine, University of Ferrara, Ferrara, Italy; ^2^ Department of Biomedical and Neuromotor Sciences, University of Bologna, Bologna, Italy; ^3^ Department of Microbiology & Immunology, Brody School of Medicine, East Carolina University, Greenville, NC, USA; ^4^ LTTA Center, University of Ferrara, Ferrara, Italy

**Keywords:** NUP214-ABL1, PI3K/Akt/mTOR signaling, T-Acute Lymphoblastic Leukemia, targeted therapies, autophagy

## Abstract

Philadelphia chromosome-positive (Ph+) Acute Lymphoblastic Leukemia (ALL) accounts for 25–30% of adult ALL and its incidence increases with age in adults >40 years old. Irrespective of age, the ABL1 fusion genes are markers of poor prognosis and amplification of the NUP214-ABL1 oncogene can be detected mainly in patients with T-ALL. T cell malignancies harboring the ABL1 fusion genes are sensitive to many cytotoxic agents, but up to date complete remissions have not been achieved. The PI3K/Akt/mTOR signaling pathway is often activated in leukemias and plays a crucial role in leukemogenesis.

We analyzed the effects of three BCR-ABL1 tyrosine kinase inhibitors (TKIs), alone and in combination with a panel of selective PI3K/Akt/mTOR inhibitors, on three NUP214-ABL1 positive T-ALL cell lines that also displayed PI3K/Akt/mTOR activation. Cells were sensitive to anti BCR-ABL1 TKIs Imatinib, Nilotinib and GZD824, that specifically targeted the ABL1 fusion protein, but not the PI3K/Akt/mTOR axis. Four drugs against the PI3K/Akt/mTOR cascade, GSK690693, NVP-BGT226, ZSTK474 and Torin-2, showed marked cytotoxic effects on T-leukemic cells, without affecting the NUP214-ABL1 kinase and related pathway. Dephosphorylation of pAkt and pS6 showed the cytotoxicity of these compounds. Either single or combined administration of drugs against the different targets displayed inhibition of cellular viability associated with a concentration-dependent induction of apoptosis, cell cycle arrest in G0/G1 phase and autophagy, having the combined treatments a significant synergistic cytotoxic effect. Co-targeting NUP214-ABL1 fusion gene and PI3K/Akt/mTOR signaling pathway could represent a new and effective pharmacological strategy to improve the outcome in NUP214-ABL1 positive T-ALL.

## INTRODUCTION

T-cell acute lymphoblastic leukemia (T-ALL) is an aggressive malignancy characterized by proliferation of thymocytes at various stages of development [1]. This disease is reported in 10–15% of children and 25% of adult ALL patients, with a significant percentage of resistance to chemotherapy and an extremely poor prognosis in case of relapse [2, 3]. Nearly 8% of T-ALL patients harbor the ABL1 tyrosine kinase gene fusion [4]. Among these fusion genes, NUP214-ABL1 is the most frequent and highly specific for T-ALL whereas BCR-ABL1 and ETV6-ABL1 are very uncommon in T-ALL and are more frequently associated with other hematologic malignancies [1]. ABL1 is reported to be fused to the BCR gene in chronic myeloid leukemia (CML) and in precursor B-cell acute lymphoblastic leukemia as a consequence of the Philadelphia translocation t(9;22) (q34;q11). ABL1-fusion proteins are involved in the pathogenesis of T-ALL despite the fact that they are infrequent in this hematological malignancy [5]. NUP214-ABL1 is a constitutively activated tyrosine kinase with oncogenic potential and has been discovered in approximately 6% of T-ALL cases. It has been found on small, cytogenetically invisible, extrachromosomal elements (episomes), associated with TLX1 or TLX3 expression and deletion of CDKN2A [6]. This and other ABL1 fusion proteins are constitutively phosphorylated, leading to an hyperactivation of survival and proliferation pathways, which can be blocked upon administration of Imatinib, a selective inhibitor of ABL1 [6–8]. NUP-214 (nucleoporin 214), an FG-repeat-containing nucleoporin, is present on the cytoplasmic side of nuclear pore complexes, and is necessary for transport between nucleus and cytoplasm, and for the regulation of the cell cycle [9]. Adults and children harboring the NUP214-ABL1 fusion gene are high-risk T-ALL patients, displaying an elevated white blood cell count, a mediastinal mass, and extramedullary involvement, often with early relapse and a poor outcome [10, 11]. NUP214-ABL1 can be easily detected by molecular biology techniques, which represent relevant tools to diagnose and monitor residual disease [12]. NUP214-ABL1 is sensitive to Imatinib and Nilotinib ABL1 kinase inhibitors and this represents an attractive therapeutic strategy for NUP214-ABL1 positive T-ALL [6, 11]. Despite this, patients under treatment with these inhibitors frequently relapse for the onset of mutations [13] and it was recently reported that a NUP214-ABL1 positive patient treated with Imatinib, after obtaining a rapid remission, fatally relapsed [12].

GZD824 is a novel tyrosine kinase inhibitor (TKI). Its antiproliferative activity was evaluated in stably transformed Ba/F3 cells whose growth is driven by native or mutant BCR-ABL1, which are mainly responsible for resistance to Imatinib as arised in clinical observations [14]. Moreover, GZD824 strongly inhibited the proliferation of human leukemia cells harboring BCR-ABL1, including K562 and KU-812 CML cell lines as well as SUP-B15 B-ALL cells, with IC_50_ values in the nanomolar range [14]. Interest is growing in multi-component targeted therapy: the combined administration of several drugs is an effort to overcome drug resistance and to improve clinical outcome. A constitutively active PI3K/Akt/mTOR signaling pathway has been reported in many types of solid and blood tumors, including T-ALL, where it causes a poorer prognosis and adversely affects the response to therapeutic treatments [3, 15]. The PI3K/Akt/mTOR signaling pathway is responsible for T-ALL survival and drug-resistance and could be targeted by small molecules inhibitors (SMIs) [3]. PI3K/Akt/mTOR inhibitors are under development for clinical use as single agents or in combination with standard chemotherapy for T-ALL treatment [16]. Aim of this study was to analyze for the first time the efficacy of Imatinib, Nilotinib and GZD824 alone and in combination with several PI3K/Akt/mTOR inhibitors in three T-ALL cell lines, ALL-SIL, PEER, and BE-13 harboring NUP214-ABL1 fusion protein. Such a combination could provide a new therapeutic option to overcome the mechanism of resistance to TKI treatment of NUP214-ABL1 positive cells. The combination of these PI3K/Akt/mTOR inhibitors with BCR-ABL1 inhibitors significantly decreased cell viability of T-ALL cells, induced cell cycle block in G0/G1, apoptosis and autophagy. These findings provide the rationale for a new promising treatment for T-ALL patients harboring the rare NUP214-ABL1 fusion gene.

## RESULTS

### PI3K/Akt/mTOR network activation in NUP214-ABL1 positive cells

**Figure 1:**
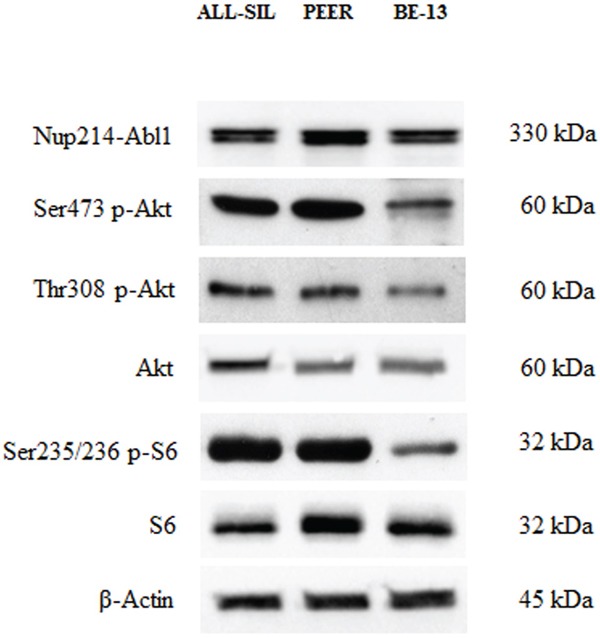
Expression and phosphorylation status of Akt and of the mTORC1 downstream target S6 in NUP214-ABL1 positive T-ALL cell lines Western blot analysis of ALL-SIL, PEER and BE-13 cell lines to detect the expression and phosphorylation levels of NUP214-ABL1, Akt and S6 proteins. Twenty-five μg of protein were blotted on each lane. Antibody to β-Actin served as loading control.

By Western Blot analysis, we verified NUP214-ABL1 presence in three T-ALL cells (ALL-SIL, PEER and BE-13) that reportedly display this fusion protein [17] (Figure 1). We then evaluated the phosphorylation level of two key proteins of the PI3K/Akt/mTOR signaling pathway in the same cell lines. All the T-ALL cells lines displayed phosphorylated Akt on both residues 473 and 308, however the expression was lower in BE-13 cells (Figure 1). Moreover, BE-13 cells showed a weaker expression of the ribosomal S6 protein phosphorylated at Ser 235/236, which is a readout of mTORC1 activity, while in ALL-SIL and PEER cells basal expression of phosphorylated S6 protein was stronger. Total Akt and S6 proteins were expressed in all cell types (Figure 1).

### Drugs targeting PI3K/Akt/mTOR display cytotoxic effects in NUP214-ABL1 positive cells

**Figure 2:**
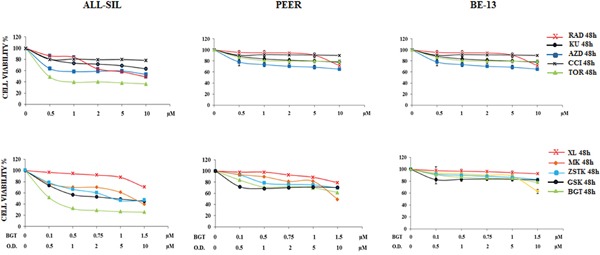
Cytotoxic activity of PI3K/Akt/mTOR inhibitors in ALL-SIL, PEER and BE-13 cell lines MTS assays of ALL-SIL, PEER and BE-13 cell lines treated with increasing concentrations of PI3K/Akt/mTOR inhibitors for 48 h. Torin-2 and BGT226 appeared to be the most effective drugs for both cell lines. SD was less than 10%. Concentration of drugs is indicated in the X axis. O.D. is the abbreviation of Other Drugs. One representative experiments of three is shown. Abbreviations: RAD001, KU0063794, AZD8055, CCI-779, Torin-2, XL-147, MK-2206, ZSTK474, GSK690693 and BGT226 were abbreviated respectively as follows: RAD, KU, AZD, CCI, TOR, XL, MK, ZSTK, GSK and BGT.

We analyzed by MTS assay the IC_50_ values of 5 different inhibitors, some of which targeted both mTOR complexes, and 5 drugs with direct inhibition of PI3K/Akt pathway in ALL-SIL, PEER and BE-13 cell lines. A different sensitivity of the cells to each drugs was observed after 48 h of treatment. In ALL-SIL, the IC_50_ values of mTOR inhibitors ranged from 0.5 to 10 μM, while PEER and BE-13 cells showed less sensitivity to the drugs, with IC_50_ values ≥ 10 μM. (Figure 2). Similar results were obtained with MTS assay of drugs against PI3K/Akt/mTOR. In ALL-SIL cells, the PI3K/mTOR inhibitor BGT226 appeared to be the most effective drug, with an IC_50_ value of 0.12 μM. The same drug was less potent in PEER and BE-13 cells, being the IC_50_ value in the range between 1.5 and 2.0 μM. All the other drugs targeting PI3K or Akt displayed an IC_50_ ranging from 3.2 to ≥ 10 μM for ALL-SIL and > 10 μM in PEER and BE-13 cells, respectively (Figure 2).

### Cytotoxic effects of Imatinib, Nilotinib and GZD824 in cell lines harboring NUP214-ABL1

**Figure 3:**
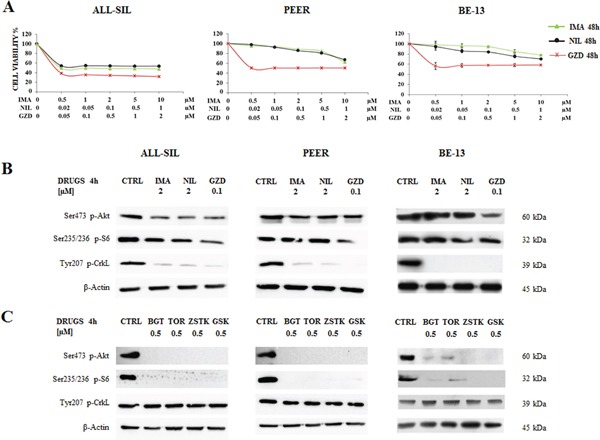
Effectiveness of Imatinib, Nilotinib and GZD824 in ALL-SIL, PEER and BE-13 cell lines **A.** T-ALL cell lines were treated with increasing concentrations of Imatinib, Nilotinib and GZD824 for 48 h. In PEER and BE-13 cells Imatinib and Nilotinib showed less potency, while the effectiveness of GZD824 was confirmed. SD was less than 10%. **B.** Western blot analysis of phosphorylated Akt, S6 and CrkL in T-ALL cell lines treated for 4 h with 2 μM Imatinib or Nilotinib or 0.1 μM GZD824. Twenty-five μg of protein were blotted to each lane. β-Actin documented equal lane loading. Imatinib, Nilotinib and GZD824 were abbreviated in IMA, NIL and GZD. **C.** Western blot analysis of phosphorylated Akt, S6 and CrkL in T-ALL cell lines treated for 4 h with 0.5 μM of BGT226, Torin-2, ZSTK474 and GSK690693. β-Actin served as loading control. BGT226, Torin-2, ZSTK474 and GSK690693 were abbreviated in BGT, TOR, ZSTK and GSK, respectively.

Given that TKIs suppress ABL1 activity, they may potentially be used in the treatment of patients with NUP214-ABL1 positive T-ALL [12]. For this reason, ALL-SIL, PEER and BE-13 cells were treated for 48 h with increasing doses of three different TKIs, Imatinib, Nilotinib, and GZD824, and analyzed by MTS assay. Results showed that in ALL-SIL cells Imatinib, Nilotinib, and GZD824 decreased cell viability and showed evident efficacy, with IC_50_ values ranging from 0.02 μM for GZD824 to 0.5 μM for Imatinib and Nilotinib. In PEER and BE-13 cells, Imatinib and Nilotinib showed less potency, while the effectiveness of GZD824 was confirmed, with an IC_50_ ≥ 0.05 μM (Figure 3A). To further confirm the efficacy of BCR-ABL1 and PI3K/Akt/mTOR inhibitors, the expression of Ser473 p-Akt, Ser235/236 p-S6 and of Tyr207 p-CrkL, a downstream substrate of NUP214-ABL1 kinase [11], were evaluated in ALL-SIL, PEER and BE-13 cells. 4 h treatment with 2 μM Imatinib or Nilotinib, or 0.1 μM GZD824 showed that the phosphorylation status of Akt on Ser473 and S6 on Ser235/236 remained almost unaffected, while the phosphorylation of CrkL was nearly abolished. This finding demonstrated the selectivity of Imatinib, Nilotinib and GZD824 (Figure 3B). At the same time, treatment for 4 h in ALL-SIL, PEER and BE-13 cells with 0.5 μM of BGT226, Torin-2, ZSTK474, and GSK690693 showed an almost complete shutdown of Ser473 Akt and S6 protein phosphorylation, while the phosphorylation status of CrkL was unchanged (Figure 3C).

### Synergism of Imatinib, Nilotinib, and GZD824 with the PI3K/Akt/mTOR inhibitors BGT226, GSK690693, ZSTK474 and Torin-2 in ALL-SIL and PEER cells

**Figure 4:**
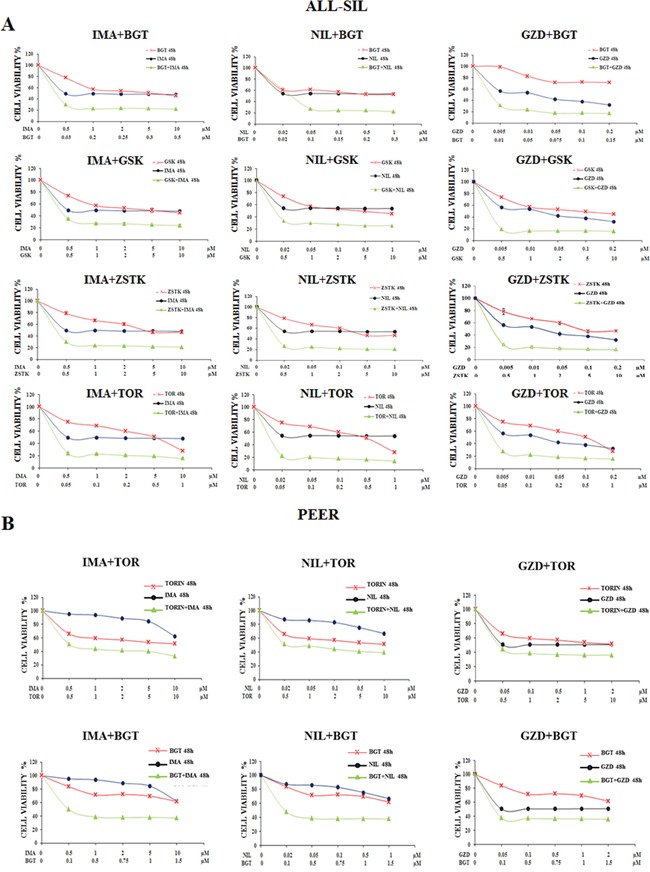
Synergism of Imatinib, Nilotinib and GZD824 with BGT226, GSK690693, ZSTK474 and Torin-2 in ALL-SIL and PEER cells **A.** MTS assays of ALL-SIL cell lines treated for 48 h with increasing concentrations of Imatinib, Nilotinib and GZD inhibitors in combination with BGT226, GSK690693, ZSTK474 and Torin-2 for 48 h. **B.** MTS assays of PEER cell lines treated for 48 h with increasing concentrations of Nilotinib, Imatinib and GZD824 inhibitors either in combination with BGT226 and Torin-2 for 48 h. Concentration of each drug is reported under the graphs. SD was less than 8%. One representative experiment of three is shown. Imatinib, Nilotinib, GZD824, BGT226, Torin-2, ZSTK474 and GSK690693 were abbreviated in IMA, NIL, GZD, BGT, TOR, ZSTK and GSK.

To better assess the effects of the simultaneous *in vitro* treatment with BCR-ABL1 and PI3K/Akt/mTOR inhibitors, we examined by MTS assay the efficacy of Imatinib, Nilotinib and GZD824 in combination with BGT226, GSK690693, ZSTK474 and Torin-2 for 48 h in ALL-SIL and PEER cells. Analysis of the results on graphs documented the existence of a significant synergism between BCR-ABL1 and PI3K/Akt/mTOR inhibitors in ALL-SIL and PEER cells as shown in Figure 4A and 4B. In PEER cells we repeated the experiments only with BGT226 and Torin-2, since these two drugs showed the most relevant synergism on the evidence of the graphs obtained.

### Increased cell cycle arrest and programmed cell death by the synergism of BCR-ABL1 and PI3K/Akt/mTOR inhibitors when compared with single administration of drugs

**Figure 5:**
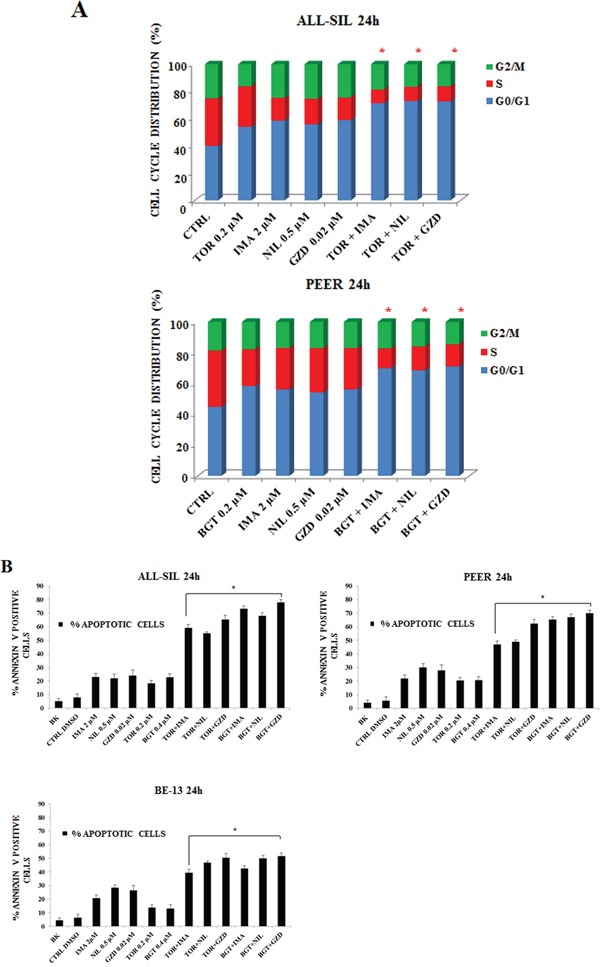
Imatinib, Nilotinib and GZD824 with BGT226 or Torin-2 induced cell cycle arrest and apoptosis in NUP214-ABL1-positive T-ALL cell lines **A.** Flow cytometric analysis of PI-stained samples in ALL-SIL and PEER cells treated with combined administration of Imatinib, Nilotinib, GZD824 with BGT226 or Torin-2 for 24 h. CTRL, control (untreated) cells. SD was less than 10%. **B.** Analysis of Annexin-V positive ALL-SIL, PEER and BE-13 cells. The analysis was performed after 24 h of treatment with single or combined drugs. Results are the mean of three different experiments ± SD. Asterisks indicate significant differences in comparison to single drug treated samples (*p< 0.05). Imatinib, Nilotinib, GZD824, Torin-2 and BGT226 were abbreviated in IMA, NIL, GZD, TOR and BGT.

To evaluate whether the drugs could influence cell cycle progression, flow cytometric analysis was performed. Imatinib, Nilotinib and GZD824 were administered alone and in combination with Torin-2 and BGT226 drugs for 24 h. These combinations augmented the G0/G1 cell cycle phase in both ALL-SIL and PEER cells, with a parallel decrease mainly in the S phase (Figure 5A).

To further analyze the mechanism of action of these drugs, Annexin-V-FITC staining was performed in all the three cell lines. Flow cytometric analysis showed that dual treatments induced a more important, statistically relevant, increase in apoptosis when compared to single drugs, with an evident synergistic effect. BE-13 cells displayed the lowest sensitivity to the drug combinations (Figure 5B).

### NUP214-ABL1 and PI3K/Akt/mTOR inhibitors induced autophagy

BCR-ABL1 is a positive regulator of autophagy, and it is deeply involved in the regulation of this process [18, 19].

**Figure 6:**
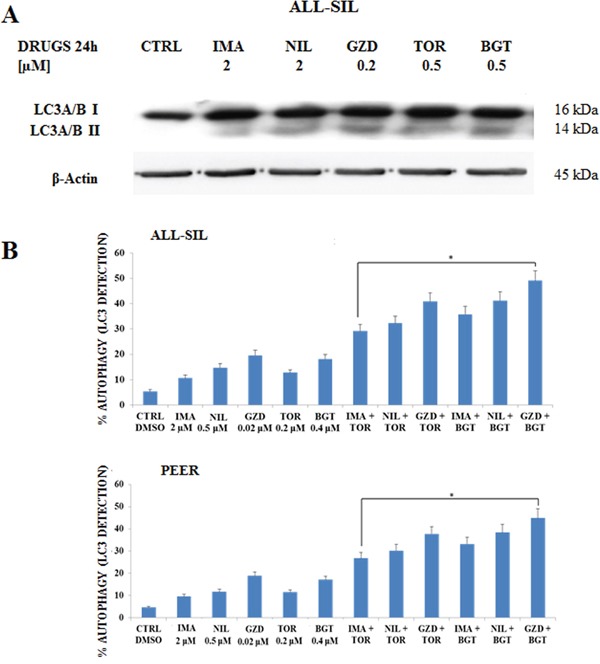
Imatinib, Nilotinib and GZD824 with BGT226 or Torin-2 induced autophagy in NUP214-ABL1-positive T-ALL cell lines **A.** Western Blot analysis in the ALL-SIL cell line treated with single administration of Imatinib, Nilotinib, GZD824, Torin-2 and BGT226 for 24 h. An increase of expression of fast-migrating (lipidated) LC3A/B after drug treatments is shown. Twenty-five μg of protein were blotted on each lane. β-Actin documented equal lane loading. **B.** Flow cytometric analysis of autophagy in the ALL-SIL and PEER cell lines treated with single or combined administration of Imatinib, Nilotinib, GZD824 with Torin-2 or BGT226 for 24 h. CTRL, control (untreated) cells. Asterisks indicate significant differences in comparison to single drug treated samples (*p< 0.05). Imatinib, Nilotinib, GZD824, Torin-2 and BGT226 were abbreviated in IMA, NIL, GZD, TOR and BGT.

To determine if the drugs could induce autophagy in NUP214-ABL1 positive leukemia cells, Western blot was performed to analyze the presence of microtubule-associated protein 1 light chain 3 LC3A/B I (non-lipidated) and its conjugated form LC3A/B II (lipidated). After 24 h of treatment of ALL-SIL cells with Imatinib, Nilotinib, GZD824, Torin-2 and BGT226, we detected an increase of LC3A/B II conjugated form (Figure 6A).

To better quantify autophagy induction, the detection of LC3A/B was performed in ALL-SIL and PEER cells by flow cytometry after 24 h of drug treatments. Results showed that all the drugs were able to induce autophagy, with a more evident effect with GZD824. Combined treatments induced a consistent, statistically important, increase in autophagy when compared to the administration of a drug alone, thus showing a synergistic effect due to the combination of different drugs (Figure 6B).

## DISCUSSION

The NUP214-ABL1 fusion gene has been described in about 6% of patients with T-ALL [10, 20]. NUP214-ABL1 discovery has emphasized the T-ALL genetic heterogeneity, but more relevant, has revealed new perspectives for targeted therapies using TKIs [21].

However, it is emerging that resistance to TKIs could develop due to activation of other signaling pathways such as the PI3K/Akt/mTOR axis [22]. T-ALL cells frequently display aberrant activation of this signaling pathway, which is due to several causes [23]. We used ALL-SIL, PEER and BE-13 T-ALL lines. All these cell lines express PTEN [24, 25] in its phosphorylated form, hence inactivated [26] (data not shown).

With the above in mind, we decided to investigate whether selective inhibitors of the PI3K/Akt/mTOR could synergize with TKIs in T-ALL cells lines displaying NUP214-ABL1 fusion protein.

GSK690693, NVP-BGT226 (BGT226), ZSTK474 and Torin-2 were used to better define their synergism with Imatinib, Nilotinib and GZD824, in terms of viability, apoptosis and cell cycle progression.

GSK690693, a small molecule ATP-competitive pan inhibitor of the pro-survival kinase Akt, preclinically tested in osteosarcoma and ALL xenografts, is now in phase I of clinical trials in sarcomas, neuroblastoma, non-glioblastoma brain tumors and lymphoma [27, 28]. NVP-BGT226 (BGT226) is an orally bioavailable dual PI3K/mTOR inhibitor in phase I/II clinical trials for therapeutic management of advanced solid tumors [29–31]. ZSTK474, a specific PI3K class I inhibitor, decreases viability and causes G1 arrest and autophagy in human breast cancer MCF-7 cells [32]. It has been tested in melanoma and prostatic tumors [33, 34] and also in leukemia models [25].

The ATP-competitive inhibitor Torin-2 is a second-generation drug directed against mTOR, which represents an encouraging therapeutic target in several human neoplasms, also for dual targeting treatment [35–37].

Our data showed that the TKIs used inhibited the proliferation of the NUP214-ABL1-positive ALL-SIL, PEER and BE-13 cells. The third generation TKI GZD824 exhibited a stronger anti-proliferative activity than either Imatinib or Nilotinib. All the TKIs fully abrogated CrkL phosphorylation at Tyr207 in NUP214-ABL1-positive cell lines. The TKIs barely inhibited the PI3K/Akt/mTOR pathway. This finding suggested that this signaling cascade is almost independent from NUP214-ABL1. Similarly, the drugs against PI3K/Akt/mTOR signaling did not affect CrkL phosphorylation.

Given the independence of these two signaling pathway, it is noteworthy that we found a synergistic effect between TKIs and SMIs that increased the efficacy of single drug administration.

ABL1 fusion protein have been often described as a cause of resistance to therapy, also related to the development of mutant clones in relapsed hematologic malignancies [38]. BCR-ABL1 independent PI3K activation has been reported as the cause of Imatinib resistance [22]. In agreement with our data, these authors showed that PI3K/Akt/mTOR activity was unaffected after treatment with Imatinib [22]. In Ph+ leukemic cell lines Nilotinib resistance was overcome by the blockade of PI3K/mTOR by using PI3K/mTOR inhibitor BEZ235 and through translational down regulation of MDM2 [39]. These observations were carried out mainly on CML cells that express the BCR-ABL1 fusion protein.

Currently, the addition TKIs to cytotoxic agents constitutes the therapeutic mainstay for BCR-ABL1-positive B-ALL patients [2].

We extended these studies to a different ABL1 fusion protein, NUP214-ABL1 and we used a third generation TKI combined with a large panel of inhibitors of the PI3K/Akt/mTOR axis.

The mechanism of resistance to ABL1 inhibitors could be observed also in NUP214-ABL1 positive T-ALL [12]. Indeed, it has been very recently reported two cases with refractory B-ALL and NUP214-ABL1 fusion [24, 40].

On the basis of the results obtained, it is justified to hypothesize that a combined therapy consisting of ABL1 TKIs and PI3K/Akt/mTOR inhibitors may offer a new therapeutic option for T-ALL patients carrying NUP214-ABL1 fusion kinase to overcome the resistance to TKIs.

We demonstrated here that autophagy is increased when NUP214-ABL1 harboring cells are treated with TKIs or SMIs, alone or in combination. Combining autophagy inhibitors with different drugs for the treatment of Ph+ leukemias may result in synergistic responses and may open new therapeutic options [41].

TKI therapy renders treatable NUP214-ABL1 subgroup of patients with T cell malignancies [12]. The TKIs investigated in this study showed different levels of efficacy with the NUP214-ABL1 ALL cell lines used: efficacy increased from first (Imatinib) to second (Nilotinib) to third (GZD824) generation.

More relevant, TKIs administration when associated with SMIs against PI3K/Akt/mTOR showed a marked synergistic effect. This association might form a novel scheme to treat TKI-resistant NUP214-ABL1 T-ALL patients.

## MATERIALS AND METHODS

### Materials

RPMI-1640 medium, fetal bovin serum (FBS), penicillin and streptomycin were from Lonza (Lonza Milano SRL, Milan, Italy). Imatinib, Nilotinib, RAD001, GZD824, GSK690693, XL-147, MK-2206, ZSTK474, BGT226, KU0063794, CCI-779, AZD8055 and Torin-2 were provided by Selleck Chemicals (Houston, TX, USA). For cell viability determination, CellTiter 96(R) AQueous One Solution Assay (MTS) was purchased from Promega (Milan, Italy). Annexin V/7-AAD, Cell Cycle, Autophagy LC3 Activation detection kits were used for the analysis with the Muse™ Cell Analyzer from Merck-Millipore (Milan, Italy). All the antibodies were from Cell Signaling Technology (Danvers, MA, USA), including the rabbit secondary antibody. The mouse secondary antibody was from Sigma Aldrich (Milan, Italy). Signals were detected with the ECL Plus reagent purchased from Perkin Elmer (Boston, MA, USA).

### Cell culture and Western blot analysis

The T-ALL cell lines ALL-SIL, PEER and BE-13 were obtained from Deutsche Sammlung von Mikroorganismen und Zellkulturen GmbH (Braunschweig, Germany). The cells were grown in RPMI 1640 medium supplemented with 20% heat-inactivated FBS, 100 units/ml penicillin and 100 mg/ml streptomycin. The cells were grown at a density of 0.5 to 2 × 10^6^ cells/ml and were incubated at 37°C with 5% CO_2_. Western Blot analysis was performed by standard methods as described elsewhere [42].

### Cell viability analysis

T-ALL cell lines were plated at 5 × 10^4^ or 2 × 10^4^ cells per well, in 96-well plates with RPMI-1640 medium supplemented with 20% FBS. The inhibitors were included in media at increasing concentrations. Viable cell number was assessed 48 h postplating by the methanethiosulfonate-based viability assay (CellTiter 96 Aqueous One Solution Reagent, Promega, Milan, Italy) as described elsewhere [43].

### Cell cycle and apoptosis analysis

Cell cycle analysis was performed using the Muse™ Cell Analyzer (Merck Millipore, Milan, Italy). In brief, after 24 h of treatment, cells were harvested, centrifuged at 300 x g for 5 min and washed once with 1X PBS. After fixing them with 70% ethanol for at least 3h at -20°C, cells were centrifuged at 300 x g for 5 min, washed once with 1X PBS and then 200 μl of Muse™ Cell Cycle reagent was added to each tube with an incubation of 30 min at room temperature in the dark. Samples were then analyzed according to the instrument protocol.

Moreover, analysis of apoptosis was performed by Annexin-V/7-AAD-Assay using the Muse™ Cell Analyzer. In brief, cells treated with increasing concentrations of the drugs, were harvested after 24 h of treatment and a 100 μl cell suspension was labeled for 20 min in the dark with the same volume of the Muse™ Annexin-V & Dead Cell reagent (Merck Millipore). Subsequently, quantitative detection of Annexin-V/7-AAD positive cells was performed with the Muse™ Cell Analyzer.

### Autophagy analysis and detection of endogenous LC3

Autophagy analysis was performed using the Muse™ Cell Analyzer. In brief, 8 × 10^4^ cells were plated in 96 well plates and treated with the different drugs for 24 or 48 h. Then, cells were harvested, treated with Autophagy Reagent A for 2-6 h, washed with Assay Buffer, incubated for 30 minutes in the dark with Anti-LC3 Alexa Fluor®555 Antibody. Samples were then analyzed according to the instrument protocol.

### Statistical evaluation

The data are presented as mean values from three separate experiments ± s.d. Data were statistically analyzed by a Dunnet test after one-way analysis of variance (ANOVA) at a level of significance of P<0.05 vs control samples [44].

## References

[1] Hagemeijer A, Graux C (2010). ABL1 rearrangements in T-cell acute lymphoblastic leukemia. Genes, chromosomes & cancer.

[2] Ferrando AA, Neuberg DS, Staunton J, Loh ML, Huard C, Raimondi SC, Behm FG, Pui CH, Downing JR, Gilliland DG, Lander ES, Golub TR, Look AT (2002). Gene expression signatures define novel oncogenic pathways in T cell acute lymphoblastic leukemia. Cancer Cell.

[3] Martelli AM, Lonetti A, Buontempo F, Ricci F, Tazzari PL, Evangelisti C, Bressanin D, Cappellini A, Orsini E, Chiarini F (2014). Targeting signaling pathways in T-cell acute lymphoblastic leukemia initiating cells. Adv Biol Regul.

[4] De Keersmaecker K, Marynen P, Cools J (2005). Genetic insights in the pathogenesis of T-cell acute lymphoblastic leukemia. Haematologica.

[5] Quentmeier H, Cools J, Macleod RA, Marynen P, Uphoff CC, Drexler HG (2005). e6-a2 BCR-ABL1 fusion in T-cell acute lymphoblastic leukemia. Leukemia.

[6] Graux C, Cools J, Melotte C, Quentmeier H, Ferrando A, Levine R, Vermeesch JR, Stul M, Dutta B, Boeckx N, Bosly A, Heimann P, Uyttebroeck A, Mentens N, Somers R, MacLeod RA (2004). Fusion of NUP214 to ABL1 on amplified episomes in T-cell acute lymphoblastic leukemia. Nature genetics.

[7] De Keersmaecker K, Graux C, Odero MD, Mentens N, Somers R, Maertens J, Wlodarska I, Vandenberghe P, Hagemeijer A, Marynen P, Cools J (2005). Fusion of EML1 to ABL1 in T-cell acute lymphoblastic leukemia with cryptic t(9;14) (q34;q32). Blood.

[8] Capdeville R, Buchdunger E, Zimmermann J, Matter A (2002). Glivec (STI571, imatinib), a rationally developed, targeted anticancer drug. Nat Rev Drug Discov.

[9] Kraemer D, Wozniak RW, Blobel G, Radu A (1994). The human CAN protein, a putative oncogene product associated with myeloid leukemogenesis, is a nuclear pore complex protein that faces the cytoplasm. Proc Natl Acad Sci U S A.

[10] Zhou MH, Yang QM (2014). NUP214 fusion genes in acute leukemia (Review). Oncol Lett.

[11] Quintas-Cardama A, Tong W, Manshouri T, Vega F, Lennon PA, Cools J, Gilliland DG, Lee F, Cortes J, Kantarjian H, Garcia-Manero G (2008). Activity of tyrosine kinase inhibitors against human NUP214-ABL1-positive T cell malignancies. Leukemia.

[12] Clarke S, O'Reilly J, Romeo G, Cooney J (2011). NUP214-ABL1 positive T-cell acute lymphoblastic leukemia patient shows an initial favorable response to imatinib therapy post relapse. Leuk Res.

[13] O'Hare T, Eide CA, Deininger MW (2007). Bcr-Abl kinase domain mutations and the unsettled problem of Bcr-Abl(T315I): Looking into the future of controlling drug resistance in chronic myeloid leukemia. Clin Lymphoma Myelom.

[14] Ren X, Pan X, Zhang Z, Wang D, Lu X, Li Y, Wen D, Long H, Luo J, Feng Y, Zhuang X, Zhang F, Liu J (2013). Identification of GZD824 as an orally bioavailable inhibitor that targets phosphorylated and nonphosphorylated breakpoint cluster region-Abelson (Bcr-Abl) kinase and overcomes clinically acquired mutation-induced resistance against imatinib. J Med Chem.

[15] Rodon J, Dienstmann R, Serra V, Tabernero J (2013). Development of PI3K inhibitors: lessons learned from early clinical trials. Nat Rev Clin Oncol.

[16] Tasian SK, Teachey DT, Rheingold SR (2014). Targeting the PI3K/mTOR Pathway in Pediatric Hematologic Malignancies. Front Oncol.

[17] Graux C, Stevens-Kroef M, Lafage M, Dastugue N, Harrison CJ, Mugneret F, Bahloula K, Struski S, Gregoire MJ, Nadal N, Lippert E, Taviaux S, Simons A (2009). Heterogeneous patterns of amplification of the NUP214-ABL1 fusion gene in T-cell acute lymphoblastic leukemia. Leukemia.

[18] Altman BJ, Jacobs SR, Mason EF, Michalek RD, MacIntyre AN, Coloff JL, Ilkayeva O, Jia W, He YW, Rathmell JC (2011). Autophagy is essential to suppress cell stress and to allow BCR-Abl-mediated leukemogenesis. Oncogene.

[19] Carew JS, Nawrocki ST, Kahue CN, Zhang H, Yang C, Chung L, Houghton JA, Huang P, Giles FJ, Cleveland JL (2007). Targeting autophagy augments the anticancer activity of the histone deacetylase inhibitor SAHA to overcome Bcr-Abl-mediated drug resistance. Blood.

[20] De Keersmaecker K, Versele M, Cools J, Superti-Furga G, Hantschel O (2008). Intrinsic differences between the catalytic properties of the oncogenic NUP214-ABL1 and BCR-ABL1 fusion protein kinases. Leukemia.

[21] Duployez N, Grzych G, Ducourneau B, Fuentes MA, Grardel N, Boyer T, Chahla WA, Bruno B, Nelken B, Clappier E, Preudhomme C (2016). NUP214-ABL1 fusion defines a rare subtype of B-cell precursor acute lymphoblastic leukemia that could benefit from tyrosine kinase inhibitors. Haematologica.

[22] Quentmeier H, Eberth S, Romani J, Zaborski M, Drexler HG (2011). BCR-ABL1-independent PI3Kinase activation causing imatinib-resistance. J Hematol Oncol.

[23] Pereira JK, Machado-Neto JA, Lopes MR, Morini BC, Traina F, Costa FF, Saad ST, Favaro P (2015). Molecular effects of the phosphatidylinositol-3-kinase inhibitor NVP-BKM120 on T and B-cell acute lymphoblastic leukaemia. Eur J Cancer.

[24] Buontempo F, Orsini E, Martins LR, Antunes I, Lonetti A, Chiarini F, Tabellini G, Evangelisti C, Melchionda F, Pession A, Bertaina A, Locatelli F, McCubrey JA (2014). Cytotoxic activity of the casein kinase 2 inhibitor CX-4945 against T-cell acute lymphoblastic leukemia: targeting the unfolded protein response signaling. Leukemia.

[25] Lonetti A, Antunes IL, Chiarini F, Orsini E, Buontempo F, Ricci F, Tazzari PL, Pagliaro P, Melchionda F, Pession A, Bertaina A, Locatelli F, McCubrey JA (2014). Activity of the pan-class I phosphoinositide 3-kinase inhibitor NVP-BKM120 in T-cell acute lymphoblastic leukemia. Leukemia.

[26] Silva A, Yunes JA, Cardoso BA, Martins LR, Jotta PY, Abecasis M, Nowill AE, Leslie NR, Cardoso AA, Barata JT (2008). PTEN posttranslational inactivation and hyperactivation of the PI3K/Akt pathway sustain primary T cell leukemia viability. J Clin Invest.

[27] Carol H, Morton CL, Gorlick R, Kolb EA, Keir ST, Reynolds CP, Kang MH, Maris JM, Billups C, Smith MA, Houghton PJ, Lock RB (2010). Initial testing (stage 1) of the Akt inhibitor GSK690693 by the pediatric preclinical testing program. Pediatr Blood Cancer.

[28] Hers I, Vincent EE, Tavare JM (2011). Akt signalling in health and disease. Cell Signal.

[29] Katanasaka Y, Kodera Y, Yunokawa M, Kitamura Y, Tamura T, Koizumi F (2014). Synergistic anti-tumor effects of a novel phosphatidyl inositol-3 kinase/mammalian target of rapamycin dual inhibitor BGT226 and gefitinib in non-small cell lung cancer cell lines. Cancer Lett.

[30] Markman B, Tabernero J, Krop I, Shapiro GI, Siu L, Chen LC, Mita M, Melendez Cuero M, Stutvoet S, Birle D, Anak O, Hackl W, Baselga J (2012). Phase I safety, pharmacokinetic, and pharmacodynamic study of the oral phosphatidylinositol-3-kinase and mTOR inhibitor BGT226 in patients with advanced solid tumors. Annals Oncol.

[31] Simioni C, Cani A, Martelli AM, Zauli G, Alameen AA, Ultimo S, Tabellini G, McCubrey JA, Capitani S, Neri LM (2015). The novel dual PI3K/mTOR inhibitor NVP-BGT226 displays cytotoxic activity in both normoxic and hypoxic hepatocarcinoma cells. Oncotarget.

[32] Wang Y, Liu J, Qiu Y, Jin M, Chen X, Fan G, Wang R, Kong D (2016). ZSTK474, a specific class I phosphatidylinositol 3-kinase inhibitor, induces G1 arrest and autophagy in human breast cancer MCF-7 cells. Oncotarget.

[33] Marone R, Erhart D, Mertz AC, Bohnacker T, Schnell C, Cmiljanovic V, Stauffer F, Garcia-Echeverria C, Giese B, Maira SM, Wymann MP (2009). Targeting melanoma with dual phosphoinositide 3-kinase/mammalian target of rapamycin inhibitors. Mol Cancer Res.

[34] Zhao W, Guo W, Zhou Q, Ma SN, Wang R, Qiu Y, Jin M, Duan HQ, Kong D (2013). *In vitro* antimetastatic effect of phosphatidylinositol 3-kinase inhibitor ZSTK474 on prostate cancer PC3 cells. Intl J Mol Sci.

[35] Watanabe T, Sato A, Kobayashi-Watanabe N, Sueoka-Aragane N, Kimura S, Sueoka E (2016). Torin2 Potentiates Anticancer Effects on Adult T-Cell Leukemia/Lymphoma by Inhibiting Mammalian Target of Rapamycin. Anticancer Res.

[36] Wang C, Wang X, Su Z, Fei H, Liu X, Pan Q (2015). The novel mTOR inhibitor Torin-2 induces autophagy and downregulates the expression of UHRF1 to suppress hepatocarcinoma cell growth. Oncol Rep.

[37] Hussain AR, Al-Romaizan M, Ahmed M, Thangavel S, Al-Dayel F, Beg S, Uddin S, Siraj AK, Al-Kuraya KS (2015). Dual Targeting of mTOR Activity with Torin2 Potentiates Anticancer Effects of Cisplatin in Epithelial Ovarian Cancer. Mol Med.

[38] De Braekeleer E, Douet-Guilbert N, Rowe D, Bown N, Morel F, Berthou C, Ferec C, De Braekeleer M (2011). ABL1 fusion genes in hematological malignancies: a review. Eur J Haematol.

[39] Ding J, Romani J, Zaborski M, MacLeod RA, Nagel S, Drexler HG, Quentmeier H (2013). Inhibition of PI3K/mTOR overcomes nilotinib resistance in BCR-ABL1 positive leukemia cells through translational down-regulation of MDM2. PloS one.

[40] Roberts KG, Li Y, Payne-Turner D, Harvey RC, Yang YL, Pei D, McCastlain K, Ding L, Lu C, Song G, Ma J, Becksfort J, Rusch M (2014). Targetable kinase-activating lesions in Ph-like acute lymphoblastic leukemia. N Eng J Med.

[41] Goussetis DJ, Gounaris E, Platanias LC (2013). BCR-ABL1-induced leukemogenesis and autophagic targeting by arsenic trioxide. Autophagy.

[42] Chiarini F, Lonetti A, Teti G, Orsini E, Bressanin D, Cappellini A, Ricci F, Tazzari PL, Ognibene A, Falconi M, Pagliaro P, Iacobucci I, Martinelli G (2012). A combination of temsirolimus, an allosteric mTOR inhibitor, with clofarabine as a new therapeutic option for patients with acute myeloid leukemia. Oncotarget.

[43] Mosmann T (1983). Rapid colorimetric assay for cellular growth and survival: application to proliferation and cytotoxicity assays. J Immunol Methods.

[44] Sparta AM, Bressanin D, Chiarini F, Lonetti A, Cappellini A, Evangelisti C, Melchionda F, Pession A, Bertaina A, Locatelli F, McCubrey JA, Martelli AM (2014). Therapeutic targeting of Polo-like kinase-1 and Aurora kinases in T-cell acute lymphoblastic leukemia. Cell Cycle.

